# An investigation into variations in the raw milk microbiota of dairy cows in Ningxia, China: effects of season, farm, parity, and health status

**DOI:** 10.1186/s12917-025-05016-z

**Published:** 2025-11-28

**Authors:** Xiu-lan Xie, Mei Cao, Shi-ying Yan, Shu Li, Hai-hui Gao, Gang Zhang, Ke-xin Deng, Jia-yi Zeng, Jian Zhao

**Affiliations:** 1 Institute of Animal Science, Ningxia Academy of Agriculture and Forestry Sciences, Yinchuan, 75002 P.R. China; 2 Key Laboratory of Biological Resource and Ecological Environment of Chinese Education Ministry, College of Life Sciences, Sichuan University, Chengdu, 610064 P.R. China; 3 Core Laboratory, School of Medicine, Sichuan Provincial People’s Hospital Affiliated to University of Electronic Science and Technology of China, Chengdu, 610072 P.R. China; 4 Key Laboratory of Ministry of Education for Protection and Utilization of Special Biological Resources in Western China, Department of Biochemistry and Molecular Biology, College of Life Sciences, Ningxia University, Yinchuan, 750021 P.R. China

**Keywords:** Raw milk microbiota, High-throughput sequencing, Season, Farm, Subclinical mastitis

## Abstract

**Background:**

The raw milk microbiota is closely associated with the physiology and pathology of the mammary gland, and plays a pivotal role in the development of offspring. The aim of this study was to examine the variability of raw milk microbiota diversity in relation to season, farm, parity, and subclinical mastitis status in Ningxia, China.

**Results:**

Raw milk samples were collected from 285 cows in Ningxia, China, followed by the exploration of microbiota using 16S rRNA high-throughput sequencing. Subsequently, all samples were categorized by season (summer vs. winter), health status (healthy vs. subclinical mastitis), farm origins (6 commercial dairy farms), and parity (primiparous vs. multiparous). Bacterial diversity, community composition, and abundance were assessed across these cohorts. Findings indicated significant variation in bacterial diversity of milk between samples collected from summer and winter. Summer samples exhibited higher bacterial richness compared to winter samples. Gut-related genera, including *Parabacteroides*, *Staphylococcus*, *Corynebacterium*_*1*, *Sphingomonas*, and *Lactobacillus* were found to be prevalent in summer milk samples. Conversely, *Escherichia-Shigella*, *Pseudomonas*, *Streptococcus*, *Psychrobacter*, *Rhizobium*, *Bifidobacterium*, and *Clostridium_sensu_stricto*_*1* were commonly observed in winter samples. The subclinical mastitis cows showed a significantly higher alpha diversity value (Chao1 and Shannon indices) compared to the healthy cows (summer). *Sporolactobacillus*, *Mycobacterium*, *Escherichia-Shigella*, and *Actionmycetaceae* were identified as biomarkers in the subclinical mastitis cows. In addition, the top 20 KEGG pathways were significantly reduced in the subclinical mastitis group compared to the healthy cows in summer, particularly in membrane transport, cell motility, and signal transduction pathways. The bacterial diversity of milk varied significantly across farms. The bacterial composition was more similar between Farms A and B, and between Farms D and F. Whereas Farm C was distinct from all others. In addition, multiparous cows exhibited greater bacterial richness than primiparous cows.

**Conclusions:**

The study indicated that the milk microbiota composition varied with season, farm, health status, and parity. These findings provide insights into the raw milk microflora in this region and can guide local farms in optimizing management and quality control measures.

## Introduction

The presence of microbiota in milk plays a crucial role in the growth and development of offspring, as well as the overall health of the maternal mammary gland [1–4]. However, certain microbes found in milk can also contribute to a shortened storage period for raw milk and potential spoilage. By utilizing both culturing methods and high-throughput sequencing, it was determined that although present in low amounts, milk contains a wide variety of microbial species [5]. Derakshani summarized the genera identified as part of the bovine milk microbiome in various studies, including *Staphylococcus*, *Streptococcus*, *Lachnospiraceae*, *Ruminococcaceae*, *Enterococcus*, *Clostridiales*, *Aerococcus*, *Acinetobacter*, *Pseudomonas*, *Stenotrophomonas*, *Prevotella*, *Bacteroidales*, *Flavobacteriaceae*, *Sphingobacterium*, *Corynebacterium*, *Bifidobacterium*, and *Propionibacterium* [6]. Due to its prevalence and impact on mammary gland health and productivity, the analysis of bovine milk microbiota had primarily centered on episodes of mastitis, the most common disease in the dairy industry [7–10]. According to a survey, *Faecalibacterium* spp., *unclassified Lachnospiraceae*, *Propionibacterium spp*., and *Aeribacillus* spp., were found to be present in healthy quarters, while *Sphingobacterium* and *Streptococcus* were associated with elevated somatic cell counts (SCC) [11]. Another investigation elucidated the compositional differences within the milk microbiota, identifying *Sphingomonas*, *Stenotrophomonas*, *Burkholderia*, and *Brevundimonas* as prevalently present in clinical mastitis, whereas healthy milk samples exhibited a dominance of *Pseudomonas* and *Psychrobacter*, and *Ralstonia* [2]. *Aerococcus urinaeequi* and non-*aureus* staphylococci in raw milk negatively correlate with *Escherichia coli* clinical mastitis [12]. The majority of commensal and environmental bacteria in milk microbiota (e.g., non-*aureus* staphylococci, *Corynebacterium* spp., and lactic acid bacteria) do not directly initiate or sustain mastitis inflammation. Dysbiosis of the gut and rumen microbiota triggers mastitis through endogenous pathways (e.g., LPS translocation and pathogenic bacterial overgrowth) and exogenous infections, while restoring microbiota homeostasis (e.g., via probiotics and short-chain fatty acids) effectively treats mastitis, highlighting the critical role of the gut/rumen-mammary axis in mastitis pathogenesis and therapy [13–15]. Notably, *Staphylococcus aureus* and *Streptococcus* spp. populations, commonly encountered in the microbiota of healthy cattle, were found to constitute a core component of the milk microbiota. These findings offered novel perspectives into the ecological dynamics of the mammary gland microbiota [6, 16].

Moreover, the composition of milk microbiota undergoes constant changes as a result of the flushing out of milk fluid and the effects of mammary gland immunity. Additionally, milk microbiota is susceptible to various influencing factors including host characteristics, environmental conditions, and sampling methods [17, 18]. The low-starch and high-fiber (LSHF) diet of cows’ milk enriched *Lachnospiraceae*, *Lactobacillus*, *Bacteroides*, and *Methanobrevibacter*; meanwhile, in the high-starch, low-fiber (HSLF) diet group, *Pseudomonas*, *Stenotrophomonas*, and *Enterobacteriaceae* were enriched [19]. The abundance of *Staphylococcaceae*, *Bacillaceae*, *Streptococcaceae*, *Microbacteriaceae*, and *Micrococcaceae* in milk was found to be influenced by seasonal variations [20]. In Farm A with a low incidence rate of subclinical mastitis, *Klebsiella*, *Escherichia-Shigella*, and *Streptococcus* were identified as primary; whereas in Farm B with a high incidence rate of subclinical mastitis, *Streptococcus* and *Corynebacterium* were determined as the main pathogens [21]. Those findings suggest that milk microbiota exhibits a high level of diversity and is influenced by various factors, thereby enhancing our comprehension of milk composition and its intricate interplay with the host and environment. Nevertheless, to date, no study has integrated these components to investigate the milk microbiota.

Although the implications of the milk microbiota in physiology and pathology are still under research, existing studies have demonstrated its crucial role in maintaining mammary gland health to combat mastitis, as well as its underlying impact on the milking performance of dairy cows. Ningxia is a leading dairy production region in China and a major base for high-quality and high-end raw materials of dairy products. The knowledge about the milk microbiota in this area remains unknown. This study employs 16 S rRNA sequencing to explore raw milk microbiota and analyze key factors related to milk production performance, including seasons, farms, healthy and subclinical mastitis cows, and parity in Ningxia, China. The objective is to offer key information regarding the raw milk microflora in this region, and to direct the production practices of local farms through optimized management and quality control measures.

## Results

**Fig. 1:**
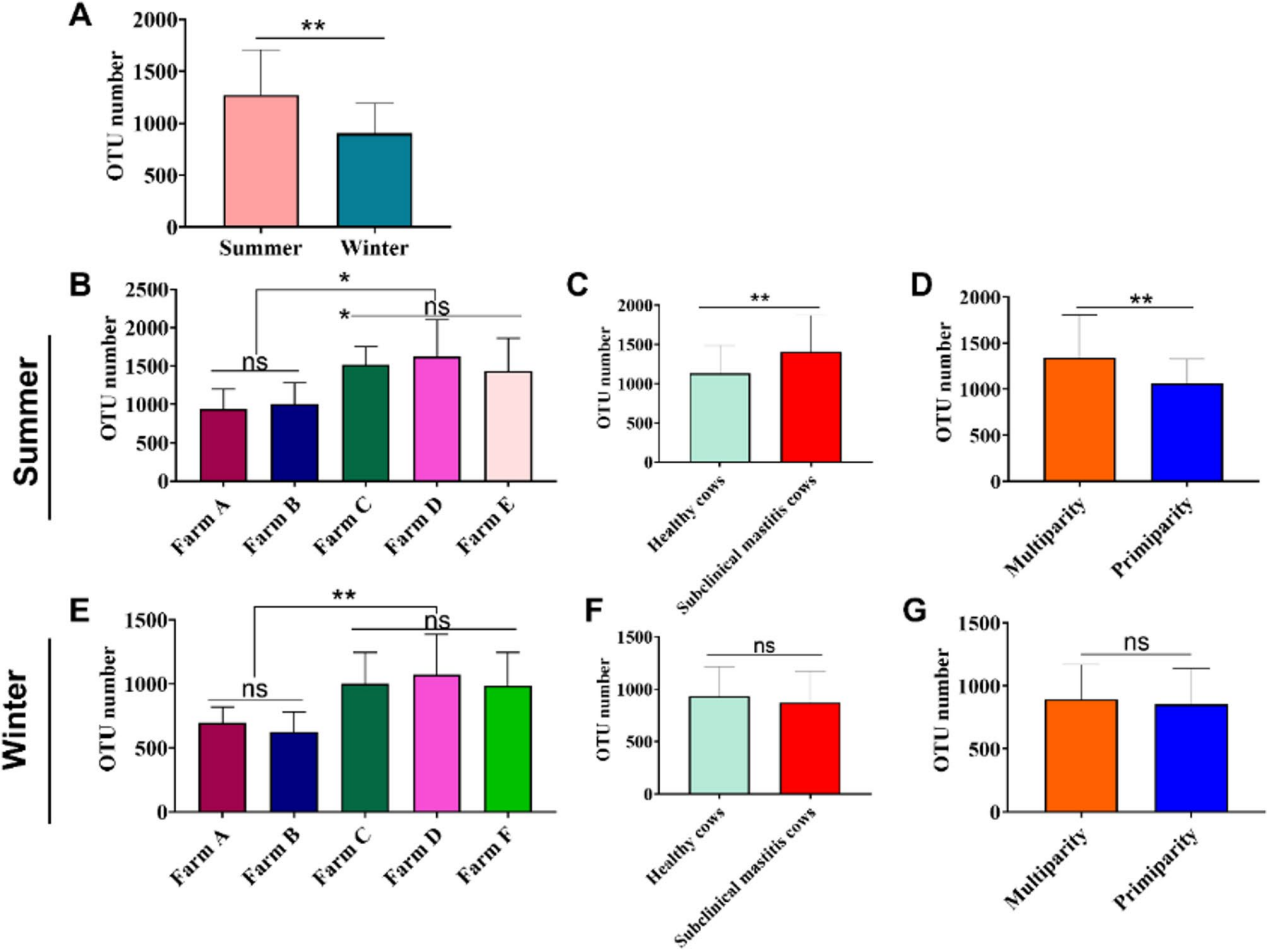
Comparison of operational taxonomic units (OTUs) derived from high-throughput sequencing of the 16S rRNA gene in raw milk. **A** OTU abundance between summer and winter samples. **B** OTU abundance across different farms in summer. **C** OTU abundance between healthy cows and those with subclinical mastitis (SCM) in summer. **D** OTU abundance between multiparous cows and primiparous cows in summer. **E** OTU abundance across different farms in winter. **F** OTU abundance between healthy and SCM cows in winter. **G** OTU abundance between multiparous and primiparous cows in winter. Data are presented as Mean ± SD, with * indicating a significant difference (*p* < 0.05), ** indicating an highly significant difference (*p* < 0.01), and ns indicating no significant difference (*p* > 0.05)

Operational taxonomic unit (OTU) abundance was significantly higher in summer than in winter (*p* < 0.01, Fig. 1A). Additionally, OTU abundance was significantly lower on Farms A and B than on Farms C, D, E, and F (Fig. 1B and E). In summer, OTU abundance was also significantly greater in cows with subclinical mastitis than in healthy cows (*p* < 0.01, Fig. 1C) and in multiparous cows than in primiparous cows (Fig. 1D). None of these patterns was evident in winter samples (Fig. 1F and G).

**Fig. 2:**
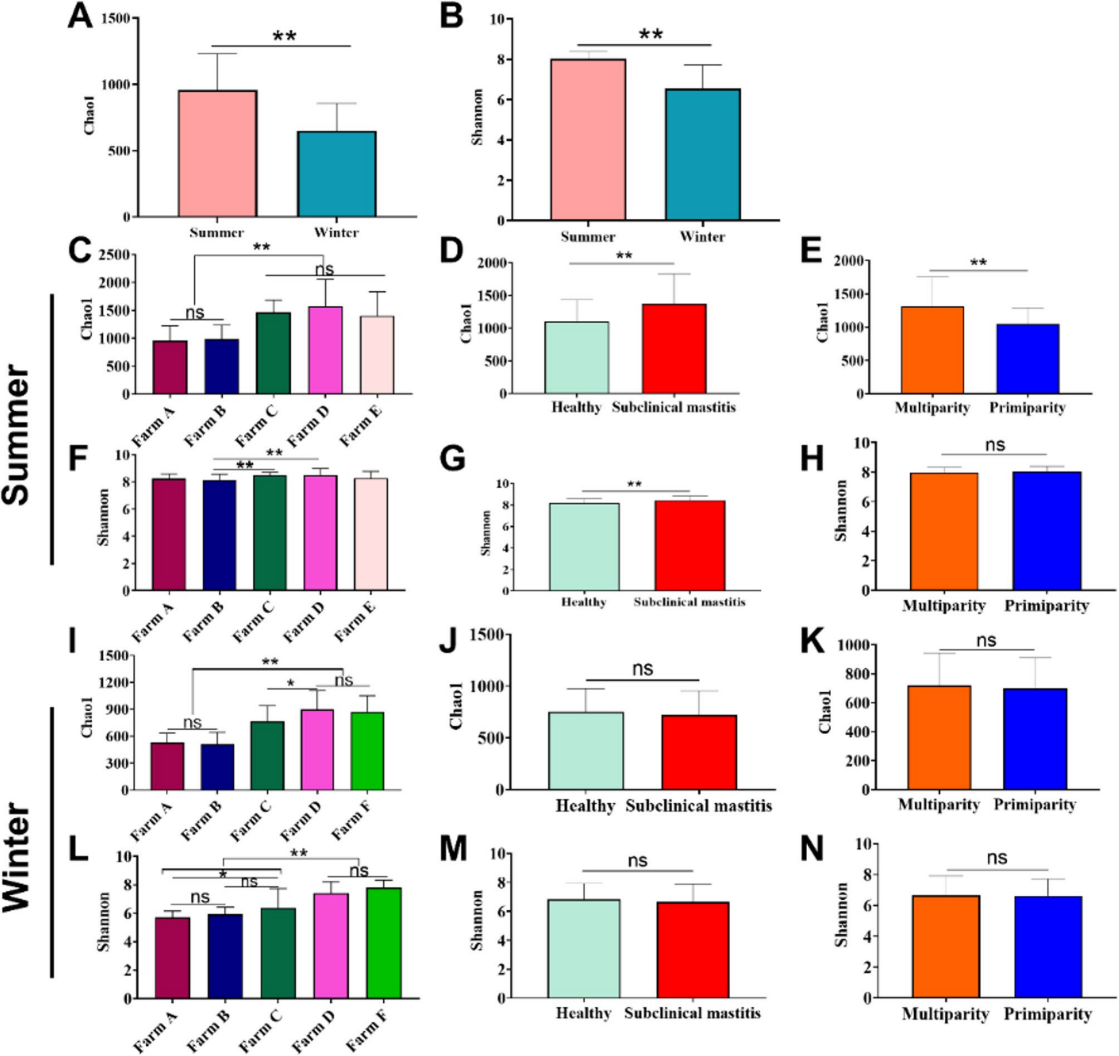
Alpha diversity analysis of raw milk microbiota. **A** The comparison of the Chao1 index between summer and winter. **B** The comparison of Shannon index between summer and winter. **C** - **E** The comparison of Chao1 index across farms, health status, and parity in summer. **F** - **H** The comparison of the Shannon index across farms, health status, and parity in summer. **I** - **K** The comparison of Chao1 index across farms, health status, and parity in winter. **L** - **N** The comparison of the Shannon index across farms, health status, and parity in winter. Data are presented as Mean ± SD, with * indicating a significant difference (*p* < 0.05), ** indicating an extremely significant difference (*p* < 0.01), and ns indicating no significant difference (*p* > 0.05)

In our study, both the Chao1 and Shannon indices were significantly higher in summer than in winter (*p* < 0.01, Fig. 2A and B). Chao1 index varied significantly among farms. In summer, values were lower on Farms A and B than on Farms C, D, and E (*p* < 0.01, Fig. 2C), while in winter, Farms A and B were lower than Farm C, D and F (*p *< 0.01, Fig. 2I). The subclinical mastitis cows displayed a higher Chao1 index than the healthy cows in summer (Fig. 2D), but not in winter (Fig. 2J). Similarly, multiparous cows had a higher Chao1 index than primiparous cows in summer (Fig. 2E), with no significant difference observed in winter (Fig. 2K). For Shannon index in summer, Farms C and D had significantly higher values than Farm B (Fig. 2F). The index was also significantly higher in subclinical mastitis cows than in healthy cows (*p* < 0.01, Fig. 2G), but no significant difference was found between multiparous and primiparous cows (Fig. 2H). In winter, Shannon index also varied by farm, with Farm D and F generally exhibiting higher values than Farms A, B, and C (Fig. 2L). No significant differences were associated with health status (Fig. 2M) or parity (Fig. 2N) during the winter season.

**Fig. 3:**
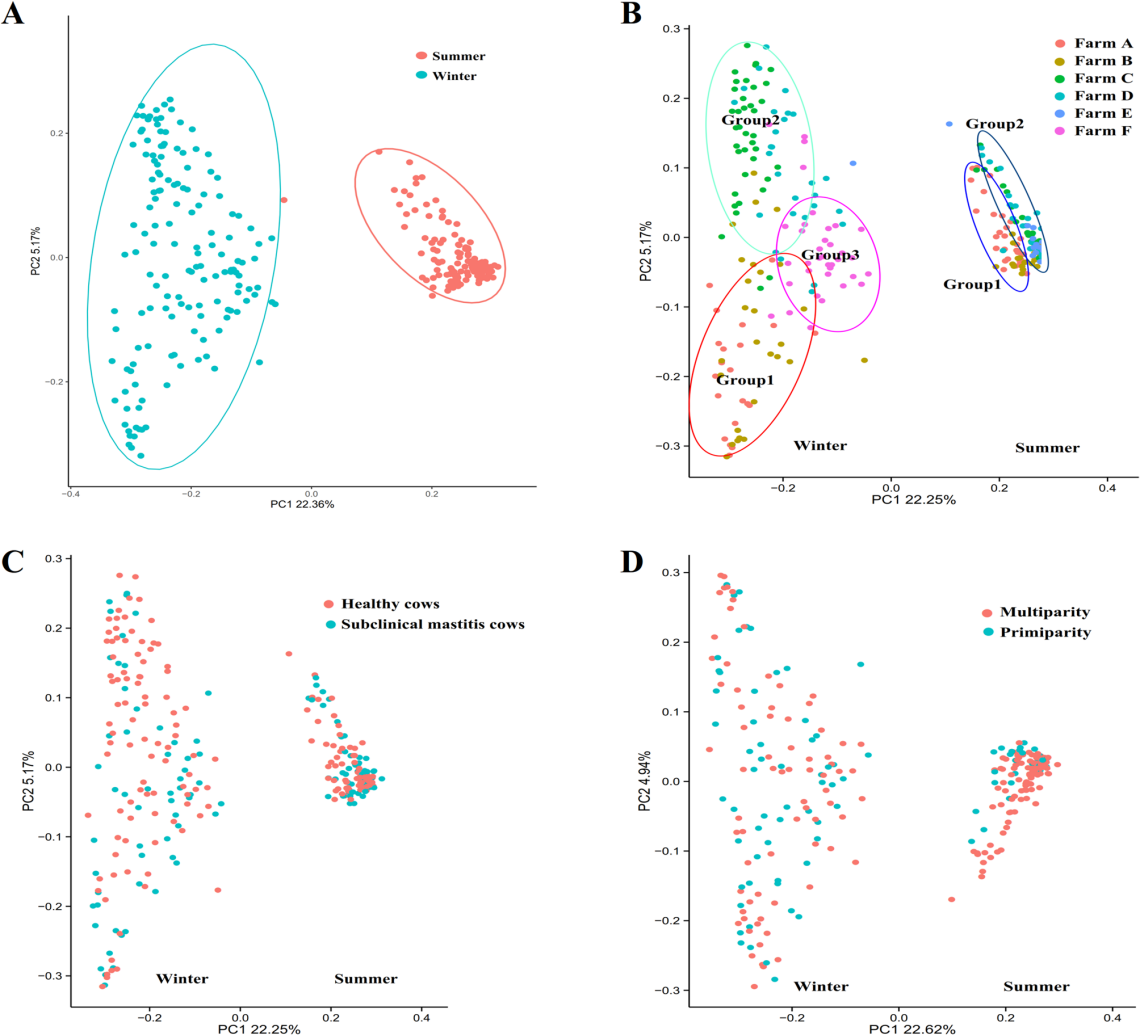
Beta diversity analysis of the raw milk microbiota was conducted using PCoA based on unweighted UniFrac distance

Beta diversity was analyzed using unweighted UniFrac principal coordinates analysis (PCoA). The PCoA plot revealed distinct microbial communities of raw milk samples collected in summer and winter, indicating that seasonality was the primary factor influencing raw milk microbial composition (Fig. 3A). Furthermore, distinct clustering was observed among farms during each season. In summer, farms grouped into two clusters: group 1 (Farms A and B) and group 2 (Farms C, D, and E) (Fig. 3B, right). In winter, the bacterial compositions formed three clusters: group 1 (Farm A, B), group 2 (Farm C, D) and Farm F (Fig. 3B, left). In contrast, no noticeable separation was observed with respect to health status or parity (Fig. 3C and D).

**Fig. 4:**
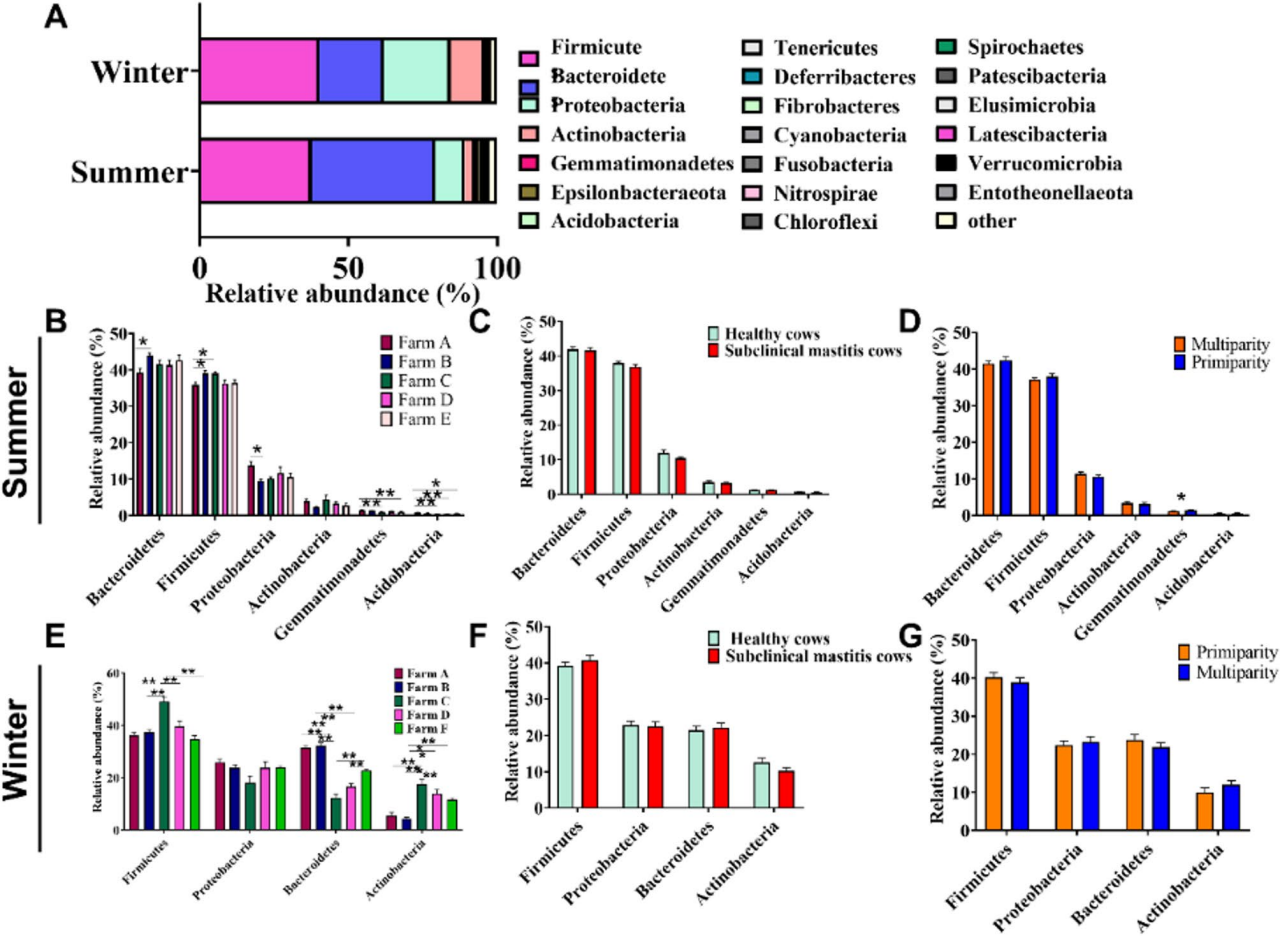
Phylum analysis of the raw milk microbiota. **A** Relative abundance of bacterial phyla (≥ 0.01%) in summer and winter samples. The "other" category represents phyla with a relative abundance < 0.01%. **B** - **D** Relative abundance of predominant bacterial phyla ( ≥ 0.5%) across farms, health status, and parity in summer. **E** - **G** Relative abundance of predominant bacterial phyla (≥ 0.5%) across farms, health status, and parity in winter. Data are presented as Mean ± SD, with * indicating a significant difference (*p* < 0.05), ** indicating highly significant difference (*p* < 0.01), and ns indicating no significant difference (*p* > 0.05)

In the summer group, the predominant phyla were *Bacteroidetes* (41.72%), *Firmicutes* (37.53%), *Proteobacteria* (11.57%), *Actinobacteria* (3.13%), *Gemmatimonadetes* (1.41%) and *Acidobacteria* (0.76%), collectively accounting for 96.13% of total sequences. In winter, *Firmicutes* (40.01%), *Proteobacteria* (22.69%), *Bacteroidetes* (21.87%) and *Actinobacteria* (11.46%) accounted for 96.03% of total sequences (Fig. 4A). In comparison to samples collected in summer, winter samples exhibited a higher abundance of *Proteobacteria* (22.69% vs. 11.57%) and *Actinobacteria* (11.46% vs. 3.13%), but a lower abundance of *Bacteroidetes* (21.87% vs. 41.72%) (Fig. 4A). Significant variations in the relative abundance of dominant phyla were observed among farms, particularly in winter (Fig. 4B and E). However, no differences were detected between the healthy and subclinical mastitis cows or between primiparous and multiparous cows, both in either season (Fig. 4C, D, F, and G). Notably, *Gemmatimonadetes* was the only phylum that was more abundant in primiparous than in multiparous cows during summer (Fig. 4D).

**Fig. 5:**
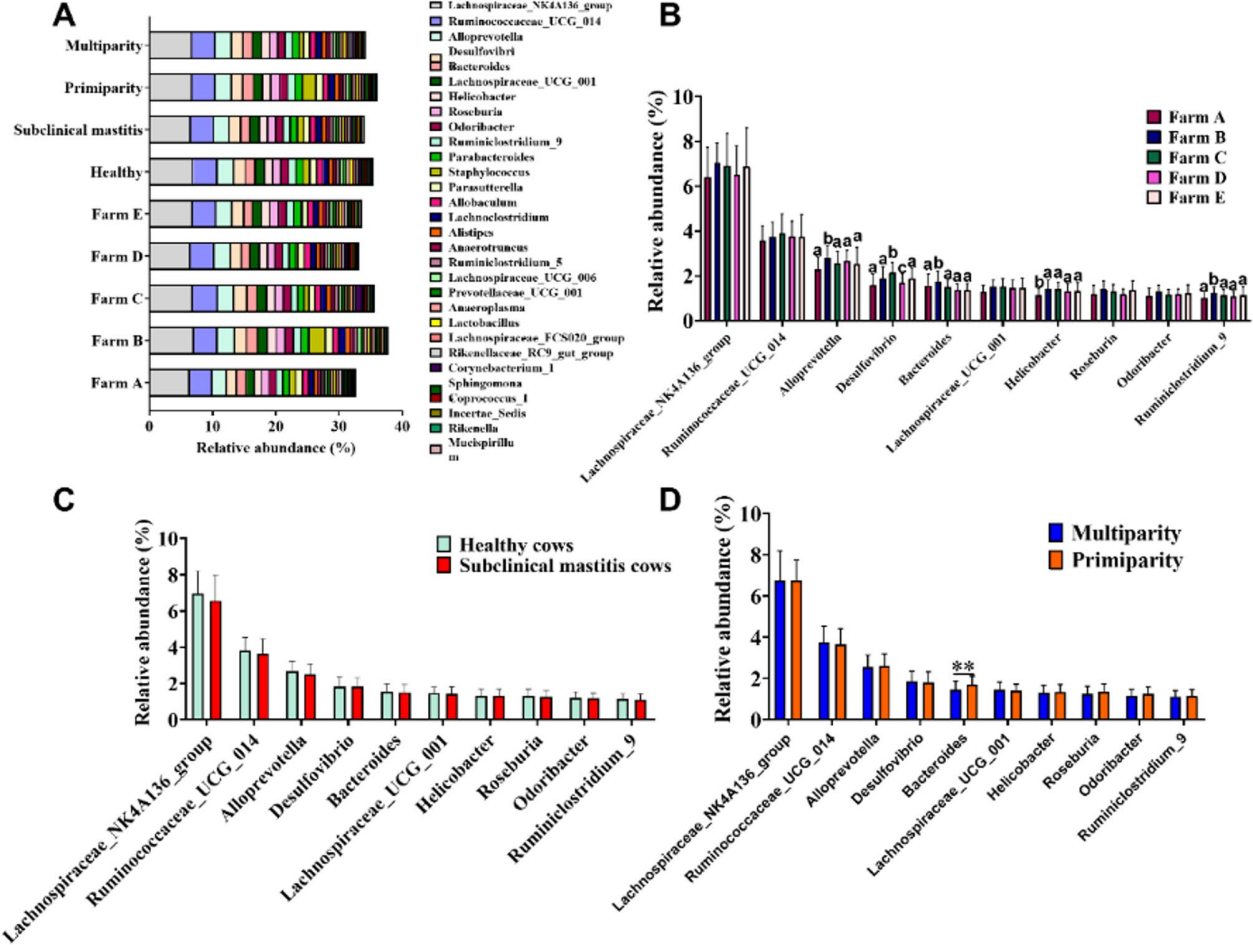
Analysis of genera in the raw milk microbiota in summer. **A** Bar chart depicting the relative abundance of the Top 30 genera across groups, with different colors representing individual genera. **B** The distribution of the Top 10 genera within five farms. **C** The comparison of Top 10 genera between healthy and subclinical mastitis cows. **D** The comparison of Top 10 genera between primiparous and multiparous cows. Data are presented as Mean ± SD, with * indicating a significant difference (*p* < 0.05), ** indicating an extremely significant difference (*p* < 0.01), and ns indicating no significant difference (*p* > 0.05)

**Fig. 6:**
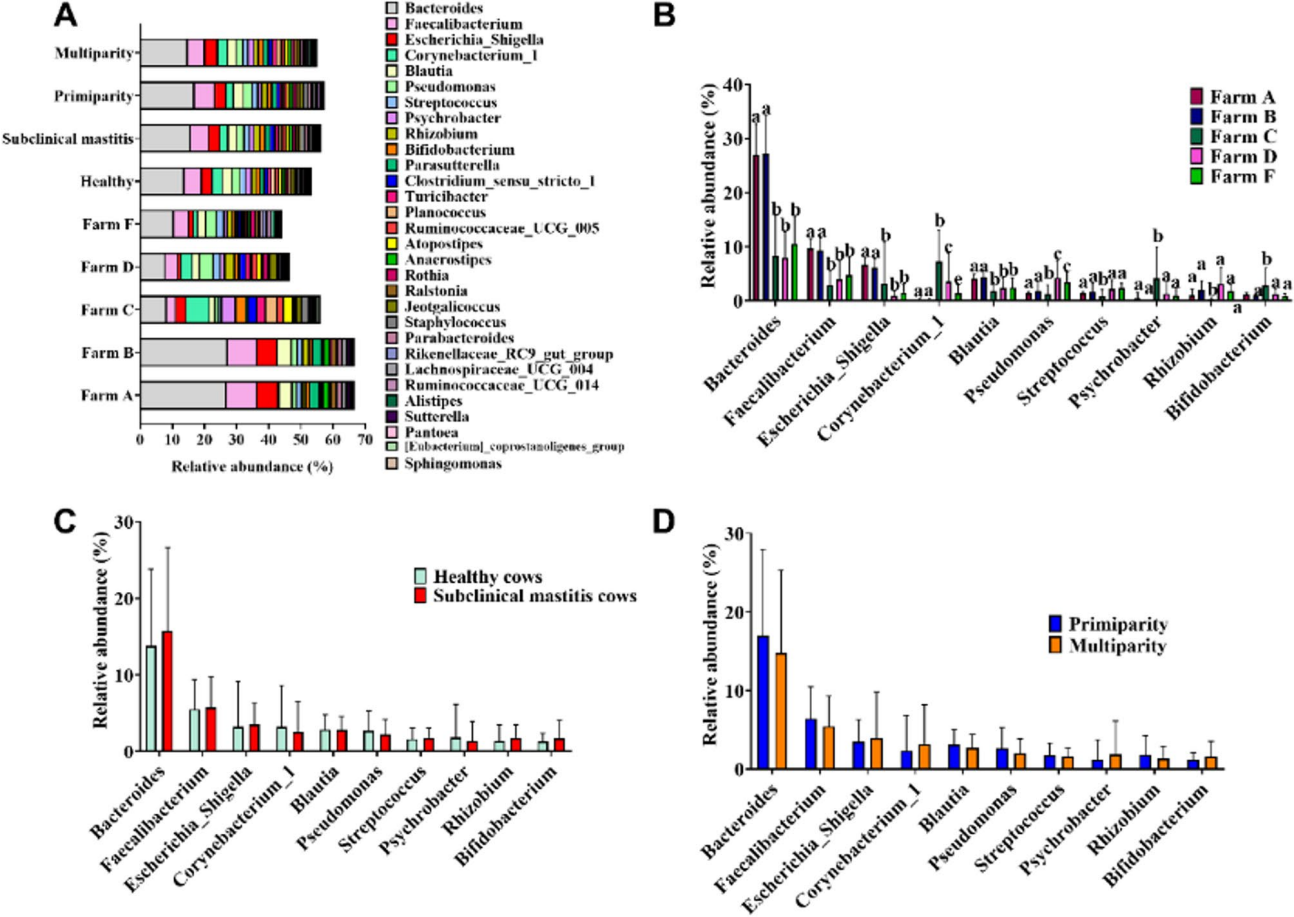
Analysis of genera in the raw milk microbiota in winter. **A** Bar chart depicting the relative abundance of the Top 30 genera across groups, with different colors representing individual genera. **B** The distribution of the Top 10 genera within five farms. **C** The comparison of Top 10 genera between healthy and subclinical mastitis cows. **D** The comparison of Top 10 genera between primiparous and multiparous cows. Data are presented as Mean ± SD, with * indicating a significant difference (*p* < 0.05), ** indicating an highly significant difference (*p* < 0.01), and ns indicating no significant difference (*p* > 0.05)

Summer samples were characterized by a high abundance of genera such as *Lachnospiraceae_NK4A136_group*, *Ruminococcaceae_UCG_014*, *Alloprevotella*, *Desulfovibrio*, and *Bacteroides* (Fig. 5A). In contrast, winter samples were dominated by *Bacteroides*, *Faecalibacterium*, *Escherichia-Shigella*, *Corynebacterium_1*, and *Blautia* (Fig. 6A). Notably, only four predominant genera (*Bacteroides*, *Corynebacterium_1*, *Parasutterella*, and *Parabacteroides*) were shared in both seasons.

We further conducted a comparative analysis of the top 10 genera changes across different cows. Variations were observed among farms, particularly in winter; however, no significant differences were found among groups: between healthy and subclinical mastitis cows, or betweenprimiparity and multiparity cows (Fig. 5A-D and 6A-D). In summer, Farm B exhibited higher abundance of *Alloprevotella*, *Bacteroides*, and *Ruminiclostridium_9* compared to the other four farms; Farm C showed higher levels of *Desulfovibrio* than the other farms; while Farm A had lower levels of *Helicobacter* compared to other farms. In winter, Farm A and B displayed higher levels of genera of *Bacteroides*, *Faecalibacterium*, *Escherichia-shigella*, and *Blautia* compared to Farm C, D, and F. Additionally, in winter, Farm C had the highest relative abundance of *Corynebacterium_1*, *Psychrobacter*, and *Bifidobacterium*, and the lowest *Pseudomonas*, *Streptococcus*, and *Rhizobium*. In summer, the primiparous group exhibited a significantly higher relative abundance of Bacteroides compared to the multiparous group (*p* < 0.01). Conversely, no significant differences were observed between healthy and subclinical mastitis cows, as well as between primiparous cows and multiparous cows in winter (*p* > 0.05).

**Fig. 7:**
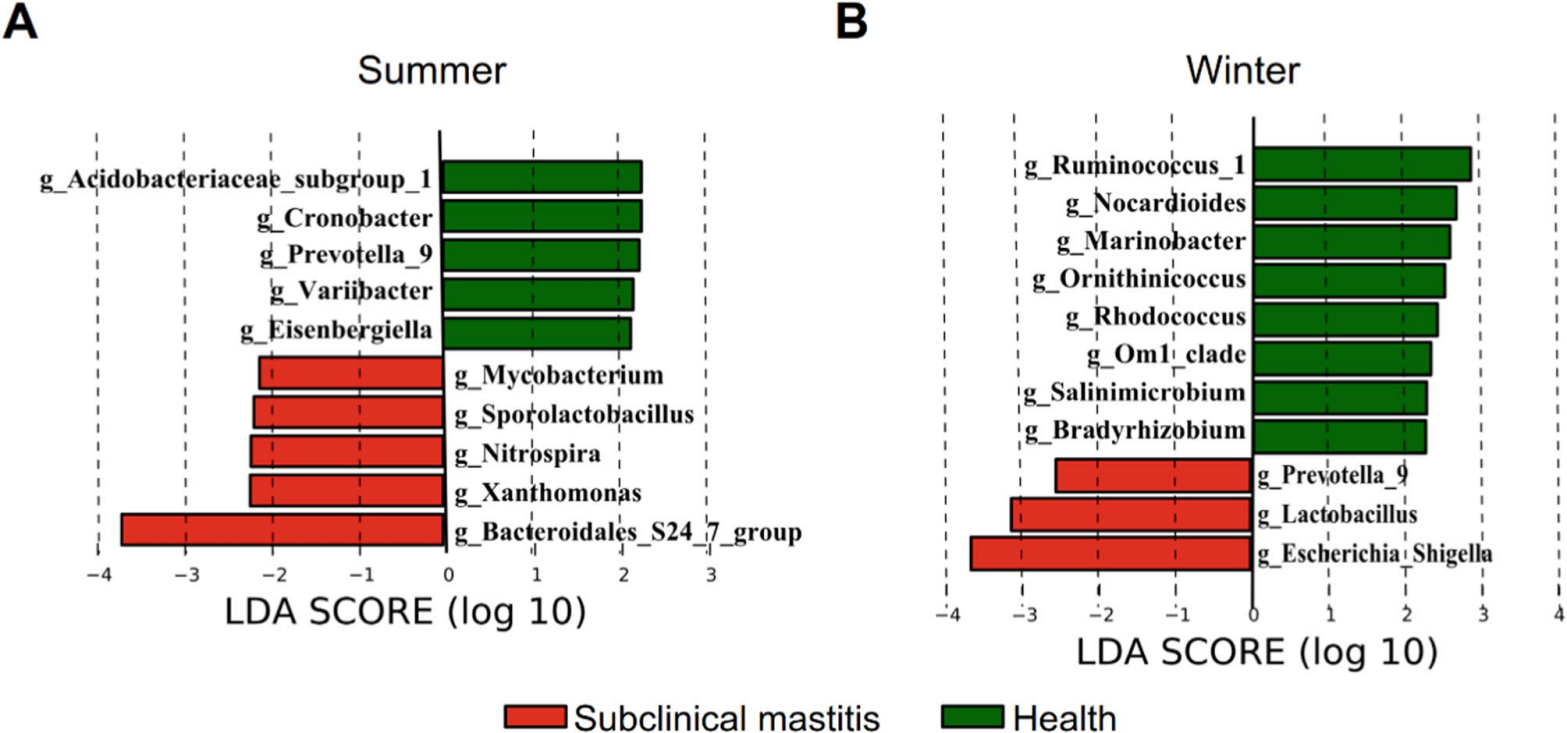
Lefse analysis of milk microbiota from healthy cows and subclinical mastitis cows

During the summer, genera such as *Acidobacteriaceae*_subgroup_1, *Cronobacter*, *Prevotella*_9, *Varibacter*, and *Eisenbergiella* were identified as biomarkers in the healthy group, while *Mycobacterium*, *Sporolactobacillus*, *Nitrospira*, *Xanthomonas*, and *Bacteroidales*_S24_7_group were identified as biomarkers in the subclinical mastitis group (Fig. 7A). In winter, *Ruminococcus*_*1*, *Nocardioides*, *Marinobacter*,* Ornithinicoccus*, *Om1_clade*, *Salinimicrobium*, and *Bradyrhizobium* were found to be biomarkers in the healthy group. Meanwhile, *Escherichia-Shigella*, *Lactobacillus*, and *Prevotella*_9 were identified as biomarkers in the subclinical mastitis group (Fig. 7B). 

**Fig. 8:**
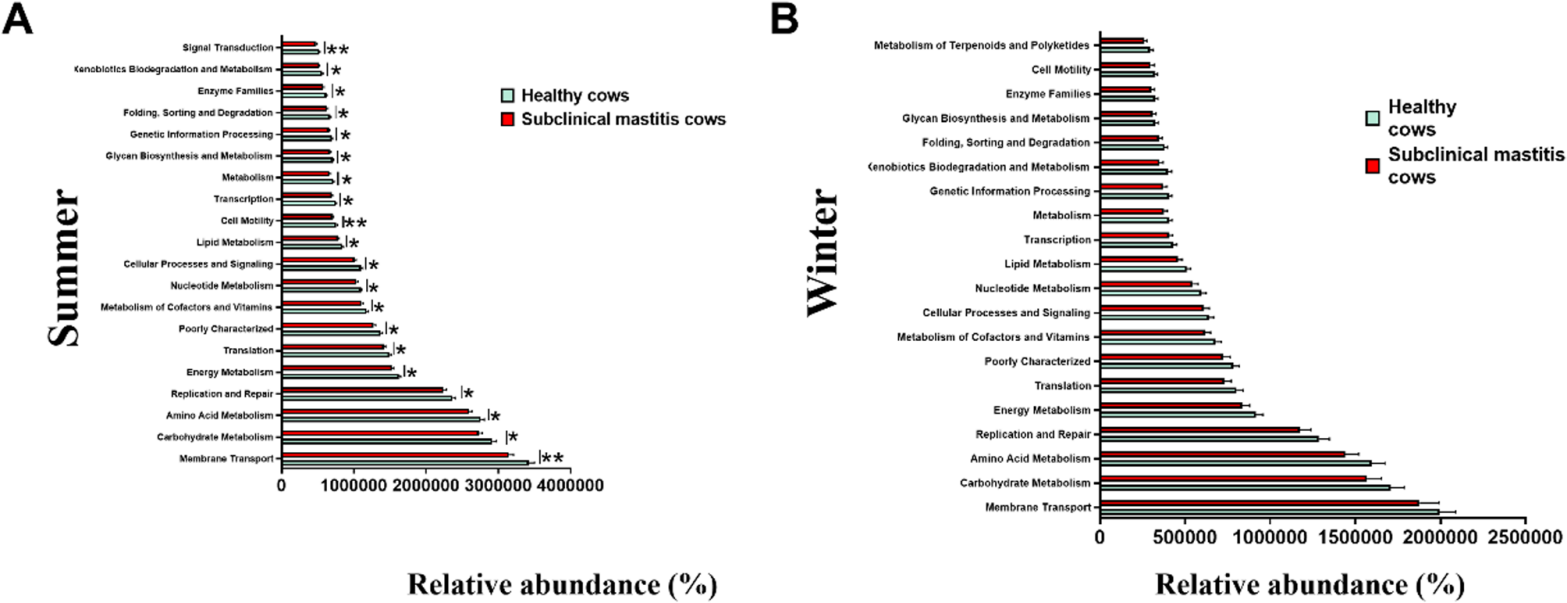
Analysis of KEGG pathways in the raw milk microflora from healthy and subclinical mastitis cows. **A **Summer. **B**.Winter. Data are presented as Mean ± SD, with * indicating a significant difference (*p* < 0.05), ** indicating an extremely significant difference (*p* < 0.01), and ns indicating no significant difference (*p* > 0.05)

A higher number of KEGG pathways were enriched in summer compared to winter. Specifically, 17 pathways exhibited significantly higher enrichment in the healthy groups compared to the subclinical mastitis group in summer (*p* < 0.05), with three pathways (cell motility, membrane transport, and signal transduction) showing highly significant differences (*p* < 0.01) (Fig. 8A). This trend was also observed in winter (Fig. 8B). Furthermore, Farm A showed some distinct differences from other farms with regards to 5 out of 20 KEGG pathways in summer; while in winter, significant changes were observed between Farms A and F, and between Farms C and F involving a total of 15 pathways. Notably, there were no differneces among the top 20 KEGG pathways between primiparous and multiparous cows both in summer and winter (data not shown).

## Discussion

Our study analyzed the cow milk microbiota across multiple variables using high-throughput sequencing.

### The discrepancy between the two seasons

The influence of seasonal variations on microorganisms is commonly attributed to temperature and humidity, which serve as the primary determinants for the majority of bacterial taxonomic variations [22, 23]. Consistent with temperature patterns, it has been demonstrated that bacterial richness tends to be lower in winter compared to summer [22, 24]. Similarly, our study revealed a significantly higher bacterial richness and diversity in milk samples collected in summer compared to winter. Furthermore, distinct differences in bacterial profiles allowed for clear differentiation between milk samples from summer and winter (Fig. 3A). The seasonal variation also extended to changes in microbiota composition. Metzger reported 11 out of the top 20 OTUs exhibited seasonal variability [23]. Nalepa’s findings indicated significant fluctuations in the species composition of raw bovine milk over a period of 22 months, with certain species/groups appearing seasonally; for example, *Lactobacillus helveticus* was more prevalent during summer, while *Lactobacillus casei* was more common during winter [25]. Nguyen observed differences in the relative abundance of genera *Staphylococcaceae*, *Bacillaceae*, *Ruminococcaceae*, *Veillonellaceae*, *Methylobacteriaceae*, and *Moraxellaceae* between the two seasons [26]. In our study, gut-associated genera such as *Lachnospiraceae_NK4A136_group*, *Ruminococcaceae_UCG_014*, and *Alloprevotella* were predominant in summer milk samples. Conversely, *Escherichia-Shigella*, *Corynebacterium_1*, *Pseudomonas*, *Streptococcus*, *Psychrobacter*, *Rhizobium*, and *Bifidobacterium* were more prevalent in winter.

Gut bacteria, including *Prevotella*, *Ruminococcus*, *Bacteroides*, *Rikenella*, and *Alistipes* have been prevalent in milk in previous studies [27–30]. *Corynebacterium*, *Streptococcus*, *Pseudomonas*, and *Psychrobacter* were found to be more commonly detected in the colder season compared to the warmer season due to their psychrophilic features [29, 31]. Additionally, *Bifidobacterium* was frequently detected in winter while *Lactobacillus* was commonly found in summer. Both of these genera are regarded as potential probiotics and are commonly present in milk samples. The reasons for the differences in their distribution remain elusive. Furthermore, several studies have documented the presence of *Rhizobium* or *Bradyrhizobium* in human milk [32, 33], goat milk [34], and cow milk [2, 35, 36]. *Rhizobium* and *Bradyrhizobium* belong to the bacterial order *Rhizobiales*. While *Rhizobia* are typically soil bacteria that form symbiotic relationships with legumes, they are not commonly recognized as gut microbes [23]. The potential explanation for their presence in milk may involve an endogenous entero-mammary pathway [4]. In addition to the aforementioned genera, *Parabacteroides* was also prevalent in our samples. Drago also discovered that Parabacteroides play a pivotal role in the bacterial network within mature milk from Italian mothers [33]. Our findings suggested that individual variations were more prevalent in winter compared to summer (Fig. 3). This may be attributed to the higher gut-associated microbiota in bovine milk during summer, with relatively stable microbiota in individuals. The KEGG pathway was utilized for analyzing known bacterial gene functions. Data from our study suggest a greater abundance of metabolism during summer compared to winter (Fig. 8), which aligns with the results reported by Li [22]. Consequently, higher bacterial abundance in summer corresponds to increased bacterial metabolism.

### The health status of the mammary gland had an impact on milk microbiota 

The original objective of our study was to compare the predominant differences in milk microbiota between healthy and subclinical mastitis cows. Enhanced richness and diversity are typically associated with improved health outcomes in systems characterized by a dense microflora, such as the gut or skin [23]. Milk from healthy cows generally exhibits greater richness and diversity of microbiota compared to that from mastitis-infected cows, even within different mammary glands of the same cow [37]. Some researchers observed disparities in milk microbiota between healthy quarters and those affected by clinical mastitis [23, 28]. Hoque also reported higher microbiota diversity and species richness in milk with mastitis conditions (including clinical mastitis, recurrent mastitis, and subclinical mastitis) than in healthy milk samples [38]. The present study revealed a significantly higher alpha diversity values (Chao1 and Shannon index) in the subclinical mastitis cows compared to the healthy cows in the summer samples (Fig. 2D and G). Furthermore, *Mycobacterium*, *Escherichia-Shigella*, and *Actinomycetaceae*, common pathogens associated with bovine mastitis [39–42], were identified as biomarkers in the subclinical mastitis cows in this study (Fig. 7A and B). In addition, our research indicated that all the top 20 KEGG pathways were significantly reduced in the subclinical mastitis group compared to the healthy group in summer, particularly in membrane transport, cell motility, and signal transduction pathways [Fig. 8A]. Increased abundance in microbial KEGG pathways indicates heightened host metabolic activity. Membrane function impairment may result in a reduced barrier function and endotoxin translocation. Abnormal signaling pathways can weaken inflammatory responses and tissue repair. These findings suggest that subclinical mastitis is characterized by impaired host metabolism. However, further validation of these results is necessary.

### The inter-farm variation in milk microbiota

In contrast to the factors influencing health status, there was limited research on the inter-farm variation of the microbiota. Pang observed that microbial diversity between two farms could be distinguished [21]. Nguyen identified differences in the relative abundance of *Pseudomonadaceae*, *Enterobacteriaceae*, and *Streptococcaceae*, *Lactobacillaceae*, *Bifidobacteriaceae*, and *Cellulomonadaceae* in milk from the two farms [26]. In addition to milk, Weese also reported high variability in fecal microbiota of calves between farms [43]. In our study, the dominant phyla were found to be similar between Farm A and B, both enriched with *Bacteroidetes*, but showing a decrease in *Actinobacteria* compared to other farms. Conversely, Farm C exhibited the highest levels of *Firmicutes* and *Actinobacteriota*, but the lowest levels of *Bacteroidetes* in winter (Fig. 4E). During summer, slight variations were observed among farms (Fig. 4B). Specifically, Farms D and E displayed similar phyla compositions, while Farm B showed higher levels of *Bacteroidetes* and *Firmicutes* as well as lower levels of *Proteobacteriota* compared to Farm A. Additionally, Farm C exhibited higher levels of *Firmicutes* than Farm A. Furthermore, significant differences were also noted in the phyla of *Gemmatimonadetes* and *Acidobacteriota* (Fig. 4B). From PCoA plots analysis, it was observed that Farms A and B exhibited more similarity in milk microbiota composition whereas Farms D and F also showed similarities. In contrast, Farm C appeared distinct from other farms (Fig. 3). In our study, all six farms are well-managed pastures participating in the dairy herd improvement (DHI) schedule. Farms A, B and C are located at the foot of Helan Mountain, providing a relatively suitable natural environment. Farms B and C are geographically closer. Farms D, E and F are situated in an open sandy land area with close proximity to each other. The herd size of Farms C is relatively small and its facilities are outdated. As a result, Farms C had the lowest milk yield. Farm D displayed higher levels of milk fat and protein but a lower yield of milk overall. Farm B, E, and F had higher milk yields than Farms A, C, and D. Farm F possessed the largest herd size, followed by Farms E, D, C, B and A (Table S1). The differences between farms can be attributed to multiple factors, including the unique characteristics of each farm, which may impact the microbial diversity in milk. Unidentified management practices could have a significant influence on the microbiota composition. For instance, during winter, Farms A and B showed enrichment of genera, such as *Bacteroides*, *Faecalibacterium*, *Escherichia-Shigella*, *Blautia*, but lower levels of *Corynebacterium_1* and *Pseudomonas*; Farm C exhibited enrichment of *Corynebacterium_1*, *Psychrobacter*, *Bifidobacterium*, and lower levels of *Rhizobium*; while Farms D and F were enriched with *Pseudomonas* and lower levels of *Escherichia-Shigella* (Fig. 6B). Given that *Pseudomonas* was prevalent in milk from healthy cows and *Escherichia-Shigella* was associated with mastitis cows, it is speculated that Farms D and F harbored a more balanced microbiota in their milk compared to Farms A, B, and C. Furthermore, our data suggested that Farms A and B were more prone to mammary gland issues related to *Escherichia-Shigella* while Farm C had higher occurrences of *Corynebacterium_1*. Overall, it is evident from our study that management practices play a crucial role in determining milk quality.

### The parity difference of the milk microbiota

Multiparous cows are at a higher risk of mastitis compared to primiparous cows [44]. Therefore, we hypothesized that parity number was an important factor influencing the milk microbiota. Lima documented variations in the bacterial composition of colostrum between primiparous and multiparous cows, with *Staphylococcus*, *Fusobacterium*, *Acinetobacter*, and *Bacteroides* being more prevalent in the colostrum of multiparous cows than in primiparous cows [45]. The colostrum microbiota of primiparous cows exhibited greater richness compared to that of multiparous cows. In contrast, our observations revealed higher bacterial richness in multiparous cows as opposed to primiparous cows (Fig. 2E). This phenomenon may be due to greater exposure to environmental microbes in the intramammary glands of multiparous cows compared to multiparous cows.

In addition to the aforementioned factors, numerous other variables have been shown to impact milk microbiota. Toscano reported disparities in the milk microbiota between Cesarean section and vaginal delivery [46]. Derakhshani observed the influence of BoLA-gene polymorphism on colostrum microbiota in dairy cows [47]. Metzger found variations in bacterial community composition among different bedding types in dairy farms [48]. Furthermore, Cabrera-Rubio demonstrated that maternal weight and mode of delivery can also affect the milk microbiota in women [49]. In summary, research on milk microbiota is still at an early stage.

Future analyses of milk microbiota in dairy cows should carefully consider the seasonal variations in sampling. Additionally, it is important to recognize the crucial role of bacteria and their metabolites in the production of high-quality fresh milk and protection of mammary gland health in dairy cattle. However, it is worth noting that current knowledge in this field is still at an early stage, as evidenced by the limited literature on the correlation between milk bacteria and their functions. Therefore, the microbiota analysis presented in this paper was primarily descriptive. It lacked comprehensive cross-sectional or longitudinal association analysis as well as correlation studies between flora and function.

## Conclusion

This study systematically reveals variations in the milk microbiota of dairy cows, demonstrating differences across seasons (summer vs. winter), health status (healthy vs. subclinical mastitis), farm environments, and parity (primiparous vs. multiparous). We identified distinct seasonal microbial patterns: summer milk was dominated by gut-associated genera such as *Parabacteroides*, *Staphylococcus*, and *Corynebacterium*_*1*, whereas winter milk showed increased abundance of potential pathogens, including *Escherichia-Shigella* and *Streptococcus*, along with cold-adapted genera (e.g., *Psychrobacter*). Notably, cows with subclinical mastitis exhibited specific biomarkers (e.g., *Sporolactobacillus* and *Mycobacterium*) and a reduction in metabolic pathways (e.g., membrane transport and signal transduction), suggesting functional impairments mediated by the microbiota during mastitis development. The observed farm-specific microbial profiles (e.g., similarities between Farms A and B and divergence in Farm C) underscore the role of farm management practices in shaping the milk microbiota. Furthermore, the higher bacterial richness in multiparous cows suggests a microbial adaptation influenced by parity. These findings not only enhance our understanding of milk microbiota ecology but also provide actionable insights for region-specific interventions. By linking microbiota variations to seasonal risks, farm management, and cow health, this study offers a scientific basis for optimizing herd health protocols, improving milk quality control, and developing targeted probiotics to mitigate mastitis in Ningxia’s dairy systems.

## Methods

### Animals and milk sample collection

The large commercial dairy farms included in the study operated the following parallel milking parlors: a 24-row system (Farms A, D, and E), a 16-row system (Farms B and C), and a 32-row system (Farm F). These systems were used to milk 1000–3000 Holstein cows thrice daily. These farms are located in the north (Farm A, N106.69, E39.12), mid (Farm B, N106.04, E38.42 and Farm C, N106.07, E38.67), and south (Farm D, N106.29, E37.75; Farm E, N106.30, E37.58; and Farm F, N106.24, E37.59) regions of Ningxia Province in China [50]. The above-mentioned six farms are situated in the dairy industry belt of the Ningxia region. Geographically, they reflect the layout of the northern (Farm A), central (Farm B and C), and southern (Farm D, E, and F) parts of Ningxia and respectively belong to six different breeding enterprises. All six investigated dairy farms in Ningxia were large-scale, intensive operations employing total mixed ration (TMR) feeding systems. To mitigate heat stress during summer, these farms implemented evaporative cooling (water spraying), maintaining barn temperatures below 28 °C. The TMR formulations were consistent in their core components: Forage base: primarily whole-plant corn silage (40–50% DM) supplemented with alfalfa hay (15–20% DM) to meet fiber and protein requirements. Concentrate mix: included ground corn, soybean meal, cottonseed meal, whole cottonseeds, and beet pulp for energy, protein, and rumen health optimization. Additives: Energy supplements: Rumen-protected fat (1–2% DM). Mineral-vitamin premix: Contained organic trace minerals (Se, Zn, Cu) and vitamins (A, D, E). Functional additives: DDGS (Distillers Dried Grains with Solubles); sodium bicarbonate (summer, 0.5% DM); mycotoxin adsorbents; and buffering agents. Farm-specific adjustments, guided by monthly Dairy Herd Improvement (DHI) records, involved fine-tuning the dietary concentrations of starch (mainly from corn), neutral detergent fiber (NDF), and metabolizable protein levels to align with lactation performance and seasonal needs. The number of lactating cows and the data regarding milk fat and milk protein in each farm are presented in Supplement Table S1. The summer milk samples were collected from August 8 to 19, 2018, and the winter samples were collected from January 14 to 23, 2019 separately. Milk samples were collected from one teat for each cow. During the summer, raw milk was collected from farms A, B, C, D, and E, totaling 135 samples; while in winter, milk samples were obtained from 150 cows in farms A, B, C, D, and F.

The sampling methods adhered to standard recommendations. In brief, the first stream of milk was discarded, and the teats were subsequently disinfected with iodine tincture for 30 s and dried with individual towels by farm veterinarians. Subsequently, the first three streams of milk were discarded and the milk samples were collected. Approximately 30 mL of milk was collected into a 50 mL sterile centrifuge tube, and temporarily stored in a storage box at 4 °C on-site and transferred to a −20 °C freezer. Following collection, the milk samples were thawed on ice and subsequently centrifuged at 12,000 rpm for 10 min at 4 °C to separate fat and cells from the whey. The pellets were then carefully collected in a 1.8 mL sterile freezing tube and stored at −80 °C for further analysis. Cows with a lactation number of 1 were defined as primiparous cows, while those with a lactation number greater than 1 (ranging from 2 to 8) were defined as multiparous cows.

All samples were collected from cows without visible signs of clinical mastitis, such as udder swelling or redness. Subclinical mastitis was diagnosed on farms by experienced veterinarians using the Lanzhou Mastitis Test (LMT) . Briefly, approximately 2 mL of milk was aseptically collected into a well of a detection disk and then mixed with 2 mL LMT reagent. The mixture was observed within 1 min, and subclinical mastitis milk was diagnosed by the presence of obvious flocculence or gelation in the mixture; milk that remained homogeneous and fluid was classified as healthy. The total sample numbers were presented in Table 1.

**Table 1:** List of sample collections

Season	Farm A	Farm B	Farm C	Farm D	Farm E	Farm F	Healthy	Sub clinical mastitis	primiparity	multiparity
Summer	30	33	25	24	23	/	67	68	35	100
Winer	21	27	38	28	/	36	73	77	58	73
Total	52	60	63	52	23	36	140	145	93	173

The symbol "/" in the table indicates that the sample was not collected, while the numerical values represent the number of raw milk samples collected from cows

### DNA extraction and library construction

DNA extraction and library construction from milk samples were performed as previously described [51]. Briefly, total DNA was extracted using a DNeasy PowerSoil Kit (Qiagen, Hilden, Germany) according to the manufacturer’s protocol. The quality and quantity of DNA were assessed using a NanoDrop 2000 spectrophotometer (Thermo Fisher Scientific, Waltham, MA, USA) and agarose gel electrophoresis. The extracted DNA was diluted to a concentration of 1 ng µL^−1^ and stored at −20 °C for subsequent processing. This diluted DNA served as the template for PCR amplification of bacterial 16S rRNA genes with barcoded primers and Ex Taq DNA polymerase kit (Takara, Dalian, China). The V3-V4 variable regions of the 16S rRNA gene were amplified with universal primers 343F and 798R (5’- TACGGRAGGCAGCAG-3’; 5’-AGGGTATCTAATCCT-3’). After two rounds of PCR amplification and purification, the amplicons were pooled in equimolar ratios for subsequent sequencing. Paired-end sequencing was performed on Illumina MiSeq platform at OE Technology Company of Shanghai.

### Sequence library analysis

The quality control process of raw data was as follows: Low-quality sequence filtering: Using Trimmomatic (version 0.35), sequences were scanned with a sliding window approach. Regions with an average base quality score below 20 were trimmed, and reads shorter than 50 bp after trimming were discarded. Paired-end sequence merging: Using Flash (version 1.2.11), qualified paired-end reads were merged into longer contiguous sequences. The maximum allowable overlap during merging was set to 200 bp to ensure accurate assembly. Strict sequence screening: The split libraries tool in QIIME (version 1.8.0) was applied to further filter sequences. Reads containing ambiguous bases (N), homopolymer repeats exceeding 8 nucleotides, or those shorter than 200 bp were removed, resulting in high-quality clean tags. Chimera removal: Potential chimeric sequences in the clean tags were identified and eliminated using UCHIME, yielding refined valid tags suitable for downstream OTU clustering and analysis. Using the Vsearch software (version 2.4.2), the high-quality valid tags obtained through quality control were classified into OTUs with a similarity of 97%, and the sequence with the highest abundance in each OTU was selected as the representative sequence of that OTU. The RDP classifier Naive Bayesian classification algorithm was adopted to annotate the representative sequences with the Silva database (version 123) to obtain the taxonomic information for each OTU.

A comparative analysis of milk microbiota differences across various groups was conducted, including summer and winter seasons, farms, health status (healthy vs. subclinical mastitis), and parities. Alpha diversity was evaluated by calculating Shannon and Chao1 indices, while beta diversity was assessed using unweighted UniFrac distance-based principal coordinate analysis (PCoA). The top 10 predominant phyla and top 30 genera among groups were compared both in summer and winter. Lefse analysis was performed to identify biomarkers in the healthy and subclinical mastitis cows. Functional prediction analysis of the 16S sequencing data was carried out using PICRUSt based on Greengenes database annotation to assess microbial gene functions in these groups. Correlations among the top 30 genera in all summer and winter milk samples were analyzed using Corrplot analysis (Spearman coefficients).

### Statistical analysis

Statistical analyses were performed using GraphPad Prism 8.0. Differences in diversity between the two groups were assessed by an unpaired two-tailed t-test. Where multiple comparisons were applicable, a one-way analysis of variance (ANOVA) was employed. Results were considered statistically significant at *p *< 0.05, and highly significant at *p *< 0.01. 

## Data Availability

The data supporting the findings of this study are openly available in the National center for biotechnology information (NCBI) sequence read archive (SRA) (accession numbers PRJNA680351).

## Abbreviations

SCC: Somatic cell counts
PCoA: Principal coordinate analysis
OTUs: Operational taxonomic units
LMT: Lanzhou Mastitis Test
